# Ral signaling pathway in health and cancer

**DOI:** 10.1002/cam4.1105

**Published:** 2017-10-18

**Authors:** Adel Rezaei Moghadam, Elham Patrad, Elham Tafsiri, Warner Peng, Benjamin Fangman, Timothy J Pluard, Anthony Accurso, Michael Salacz, Kushal Shah, Brandon Ricke, Danse Bi, Kyle Kimura, Leland Graves, Marzieh Khajoie Najad, Roya Dolatkhah, Zohreh Sanaat, Mina Yazdi, Naeimeh Tavakolinia, Mohammad Mazani, Mojtaba Amani, Saeid Ghavami, Robyn Gartell, Colleen Reilly, Zaid Naima, Tuba Esfandyari, Faris Farassati

**Affiliations:** ^1^ Department of Human Anatomy and Cell Science University of Manitoba Winnipeg Canada; ^2^ Department of Medicine, Molecular Medicine Laboratory The University of Kansas Medical School Kansas City Kansas; ^3^ Department of Pediatrics, Columbia Presbyterian Medical Center New York New York; ^4^ Saint Luke's Hospital University of Missouri at Kansas City Kansas City Missouri; ^5^ Pasteur Institute of Iran Tehran Iran; ^6^ Research Service (151) Kansas City Veteran Affairs Medical Center & Midwest Biomedical Research Foundation 4801 E Linwood Blvd Kansas City Missouri 64128‐2226; ^7^ Ardabil University of Medical Sciences, Biochemistry Ardabil Iran

**Keywords:** Aurora Kinase, biology, cancer, cancer stem cells, Ral, therapy

## Abstract

The Ral (Ras‐Like) signaling pathway plays an important role in the biology of cells. A plethora of effects is regulated by this signaling pathway and its prooncogenic effectors. Our team has demonstrated the overactivation of the RalA signaling pathway in a number of human malignancies including cancers of the liver, ovary, lung, brain, and malignant peripheral nerve sheath tumors. Additionally, we have shown that the activation of RalA in cancer stem cells is higher in comparison with differentiated cancer cells. In this article, we review the role of Ral signaling in health and disease with a focus on the role of this multifunctional protein in the generation of therapies for cancer. An improved understanding of this pathway can lead to development of a novel class of anticancer therapies that functions on the basis of intervention with RalA or its downstream effectors.

## Ral Proteins and Their Structure

Ral (Ras‐like) GTPases are coded for by two genes (RalA and RalB) which are located on human chromosomes 7 and 2, respectively 1. These genes were first isolated from a simian B‐cell cDNA library by probing with an oligonucleotide corresponding to one of the GTP‐binding domains of Ras 2 and named according to their sequential homology with the Ras family of small GTPases 3. Although RalA and RalB are more than 85% sequence identical, they have distinct functions 1. These differences may partly stem from their distinct C‐terminal sequences, which can influence subcellular membrane localizations as well as effector interactions 4. The Ral protein is comprised of 206 amino acids and therefore is slightly longer than a Ras protein (188–189 amino acids). Eleven additional amino acids in the N‐terminal end and six additional amino acids in the C‐terminal domain have been found in the Ral proteins that are not found in Ras proteins. However, the catalytic domain (5–164) of Ral and Ras proteins is highly conserved 5, 6.

The bulk of the protein (180 residues) is a G‐domain, which includes four GTP‐binding motifs conserved in small GTPases, and an effector‐binding loop which is involved in effector interaction. Moreover, the strongest identity is seen within the N‐terminal 90 residues (98% identity) which are 100% conserved in RalA and RalB 4, 7. The first 11 amino acid residues at the N‐terminus interact with PLD or PLC*δ*1 3. These regions are called switch I (residues 41‐51) and switch II (residues 69–81) which are involved in binding to multiple targets, including RalBP1, Sec5, Exo84, and ZONAB. These two regions correspond to the largest conformational changes during GTP‐GDP cycle 5, 6. A basic/hydrophobic amino acid‐rich region that forms an amphipathic *α*‐helix is found in the C‐terminal region of RalA which has been shown to bind with calmodulin. A recent study has shown that both RalA and RalB have calcium‐dependent calmodulin‐binding sites in their C‐terminal domains as well as calcium‐independent‐binding sites in their N‐terminals 8. The highly variable regions of RalA and RalB with 50% identity are in the C‐terminal membrane‐targeting sequence ending with a CAAX tetrapeptide sequence 2, 7, 9, 10.

Both sequence and targeted mutational analysis of this variable region have indicated that there are unique phosphorylation sites for RalA or RalB which have a significant impact on subcellular localization and protein function 11, 12. Several studies have reported that the two serine residues (S_194_ and S_183_) in the RalA C‐terminus and five threonine or serine residues (T_178_, S_182_, S_192_, S_193_, and S_198_) in the RalB C‐terminus are special targets for phosphorylation 10, 12, 13. For instance, RalA can be phosphorylated on serine 194 in cells by Aurora Kinase A (AKA), a mitotic kinase, and can be dephosphorylated by protein phosphatase 2A (PP2A) A*β* (also known as PPP2R2B), a tumor suppressor 13, 14, 15. Recent research has shown that phosphorylation of RalA on S194 can favor the binding of RalA to RalBP1 over Sec5 and Exo84 11. RalB is phosphorylated at Ser198 by protein kinase C (PKC). The Ser198 site in RalB conforms to a motif recognized by PKC, and the mutation of this Serine to Alanine reduces PKC phosphorylation to approximately 70%, with the other 30% of phosphorylation presumably occurring at other sites. The phosphorylation of Ser198 is important for anchorage‐independent cell growth, cell motility, and actin cytoskeletal organization for in vivo bladder cancer tumorigenicity driven by active RalB 12. These findings demonstrate that despite their extensive sequence identity, these Ral proteins have different cellular functions.

## Regulation of Ral Activity

**Figure 1:**
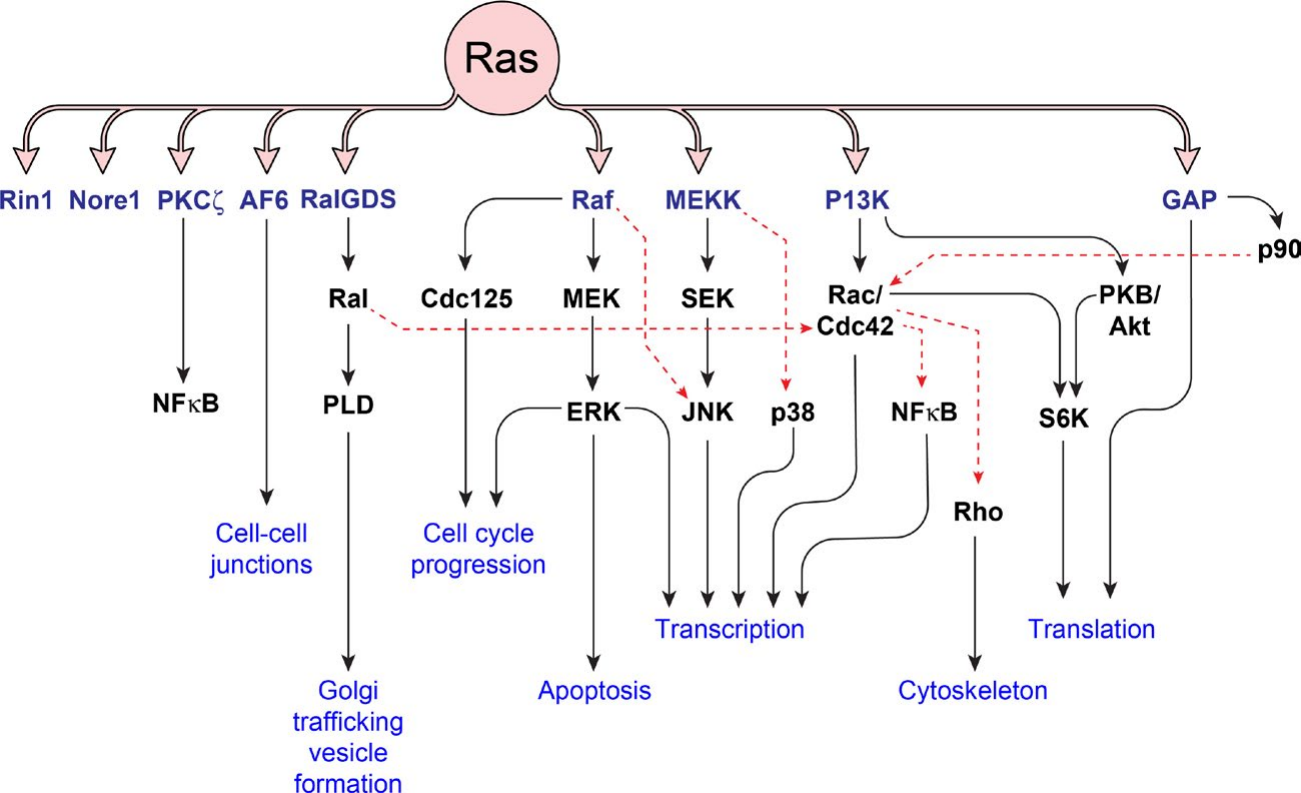
Downstream effectors of Ras pathway including Ral pathway and their biological roles. A series of downstream effectors of Ras exert a myriad of biological effects which in case of overactivation can contribute to progression toward neoplasia. Ral pathway is activated by RalGDS a member of RalGEF family. Although RalGDS shares sequence homology with Ras‐GEFs, it does not show any affinity for Ras. Activation of PLD by Ral results in generation of a series of second messengers such as phosphatidic acid, lysophosphatidic acid, and diacylglycerol. Ral also activates Cdc42 in a cross‐talk with the PI3K pathway.

The activation of Ral is initiated through the recruitment of upstream Ras‐dependent or Ras‐independent signaling. Investigations in follicular epithelial cells from human thyroid identified Ral as an effector of oncogenic mutant Ras 16. In mammals, there are three recognized downstream pathways for Ras signaling (i.e., Raf, PI3K, and a Ral‐GEFs family including RalGDS, Rgl1, Rgl2/Rif, and Rgl3), two of which seem to play an important role in the regulation of Ral activity 17, 18. PIP3 is mediated upon activation of PI3K and subsequently phosphorylates AKT by recruiting PDK1 in the plasma membrane, which can then induce the activation of RAL–GEF (Ral‐GDS) 19, 20. Activated Ras is also linked directly to the binding and recruiting of RalGEFs to RalA and RalB small GTPases 21 (Fig. 1). Ral proteins can also be activated without Ras via RalGPS1 and RalGPS2 22, 23. RalGPS2 is required for RalA activation, whereas RalGPS1 has been shown to be involved in the regulation of RalB 24. Data show that activation of RalA is regulated by Ca^2+^ release from intracellular stores 25 and calmodulin binding 26 in a non‐Ras‐dependent manner. The results suggested by others 27 raise the possibility that AKA is phosphorylated by the Ca2+–calmodulin‐dependent pathway that may control the activation of RalA in cancer and normal cells. The AKA not only triggers RalA activation but it may also play a role in regulating RalBP1 activity 15. We have recently investigated the outcome of inhibition of AKA on RalA activation and malignant phenotype of ovarian and liver cancer cells 28, 29. Ral‐GDS can also be activated in a non‐Ras‐dependent manner by cleaving the inactive *β*‐arrestin–Ral‐GDS protein complex in response to formyl‐Met‐Leu‐Phe (fMLP) receptor stimulation 30. Nitric oxide (NO) is another molecule that appears to be important in both Rap1 and Ral activation 31. It is mentioned that both LPA1 and LPA2 receptors are associated with regulation of the small GTPase and RalA. Furthermore, LPA triggers RalA activation through LPA1 32. Interestingly, G protein‐coupled receptors (GPCRs) have been shown to stimulate phospholipase C and function in a Ral‐dependent manner 33, 34. Insulin‐stimulated pathways have been identified to be crucial for Ral activation. Insulin leads to a robust activation of PI3 kinase which further stimulates AKT kinase activity which is able to inhibit the interaction of Ral‐GAP complex (GAP) and RalA, resulting in the increase of RalA expression 35. ERP57 is known to be an inhibitory regulator of the RalA signaling pathway which binds to and inhibits the exchange of GDP‐inactive to GTP‐bound form 36.

## Ral Effector Proteins

**Figure 2:**
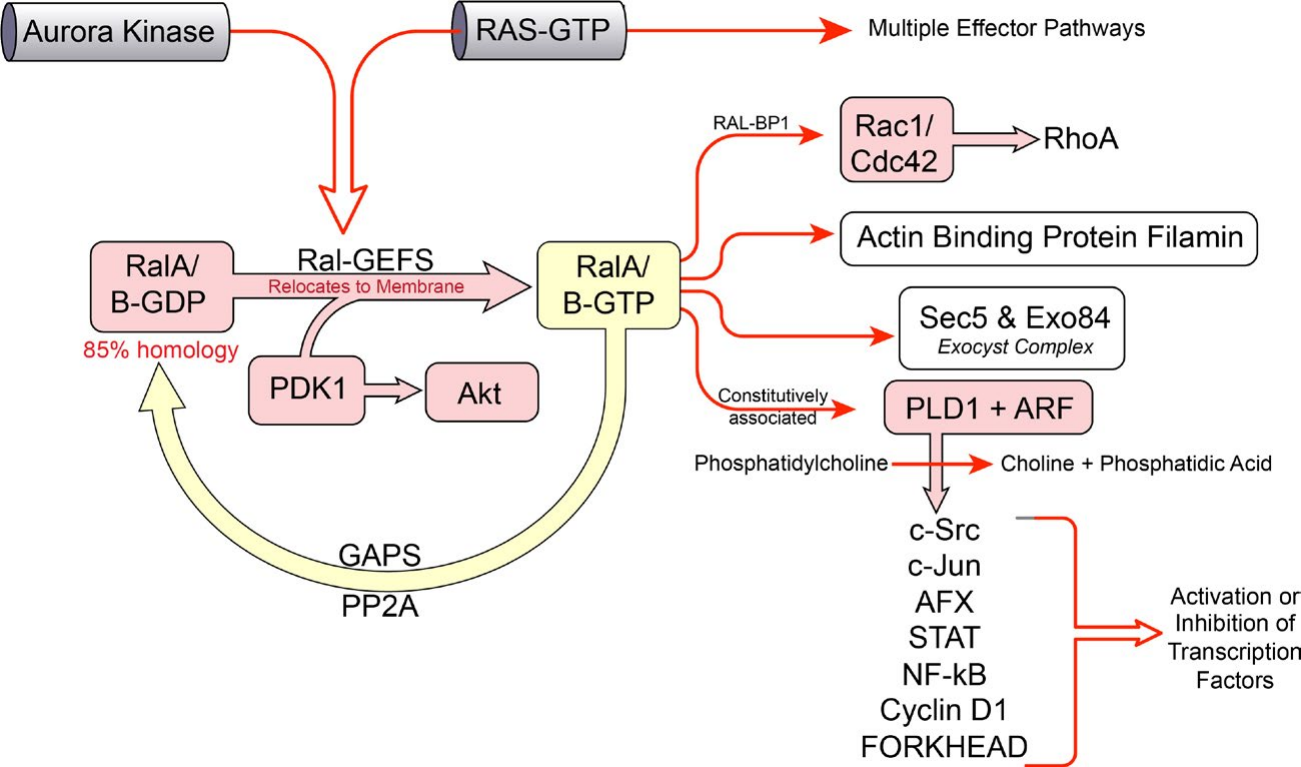
RalA/B effectors. Once in GTP‐bound (active) form, Ral proteins activate a collection of different effectors. Effectors such as ZONAB, Sec5, Exo84, Filamin, and RalBP1 are involved in a variety of biological effects such as gene transcription, cell proliferation, cell survival, and actin organization. PLD is involved in production of second messengers influencing activation status of a variety of transcription factors such as STAT3, ATF‐2, FOXO4, NFAT, c‐Jun.

Activated GTP‐bound Ral (Ral‐GTP) was found to interact directly with a vast spectrum of downstream effectors and regulate their activation 37 (Fig. 2). In a yeast two‐hybrid screen, RalBP1 (RLIP76), a 76 kDa Ral‐binding protein, was discovered as the first Ral effector, which has regulatory function in the Ral network 38. RalBP1 negatively regulates the Rho family of small GTPases including Rac‐1 and Cdc42. Morphogenesis, actin dynamics, nuclear responses, and cell cycle progression are controlled by Rac‐1 and Cdc42 38, 39, 40. RalBP1, through a conserved Ral‐binding domain in its C‐terminal, interacts with the activated form of RalA and RalB 38, 41 and may also form a complex with the tyrosine‐phosphorylated proteins, Reps1 (RalBP1‐associated Eps homology (EH) domain protein 1) and Reps2/POB1 (partner of RalBP1) 42, 43. Both of these proteins contain EH domains which exist in proteins involved in endocytosis 44. Reps1 binds to Rab11‐FIP2 via its EH domains 45, while POB1 binds to either Epsin or Eps15 to make a complex with AP2‐clathrin 46. Reps1 can also bind to the adaptor proteins like Crk and Grb2 17 and may stimulate epidermal growth factor (EGF) receptor activation and lead to Ral‐GTPases activation 42. It has been shown that Rab11‐FIP2 binding to Reps1 is involved in endocytosis, while the binding of POB1 to Epsin inhibits receptor‐mediated endocytosis by phosphorylation of Epsin in mitosis 46, 47. Epsin interaction with RalBP1 can regulate its function in Arf6/Rac1‐dependent pathways, leading to GTPase activation and ultimately promotes cell migration and invasion 48. *μ*2, a subunit of the heterotetrameric clathrin adaptor (AP‐2 complex), is shown to make a complex with RalBP1. It is proposed that *μ*2 of the AP2 complex, like POB1 or Reps1, is involved in clathrin endocytosis by interacting with RalBP1 49, 50. RalBP1 also interacts with ARIP2 which regulates the endocytosis of activin by ActRIIs‐mediated internalization through the Ral/RalBP1‐dependent pathway 51. The interaction between RalBP1 and mammalian heat shock factor 1 (HSF1) results in the ability to regulate expression of heat shock genes in times of stress 52.

The binding of CDK1 to RalBP1 induces its translocation to chromosomes that contribute to terminating mitosis 53. RalBP1, as a glutathione‐conjugate transporter and an antiapoptotic protein, is necessary for chemical carcinogenesis, drug resistance, cancer progression, and spontaneous and experimental metastasis models 54, 55, 56. The inhibition of transport activity of RalBP1 has been found to be effective for cancer cell survival by playing a role in stress defense mechanisms 57. Several studies also revealed that RalBP1 inhibition could cause regression of lung, colon, prostate, kidney, and melanoma cancer xenografts 37, 57, 58.

Other components of the octameric exocyst complex are Sec5 and Exo84 59, which are implicated in tethering post‐Golgi secretory vesicles to the plasma membrane before exocytic fusion (Spiczka et al. 2008). Sec3 and Exo70 associate with the exocyst subunits, thereby causing the maintenance of epithelial cell polarity, cytokinesis, cell migration, and tumor cell invasion 60. Filamin A is another effector that acts via RalA. The fact that the activation of RalA may recruit cytoskeletal filamin prior to filopodial protrusion has been shown to be responsible for the formation of filopodia 61.

Ral proteins associate with phospholipase C‐*δ*1(PLC *δ*1), and their binding is enhanced in the presence of Ca^2+^ 34. Binding of Ral to PLC *δ*1 is not nucleotide‐dependent and might stimulate G‐protein‐coupled receptors as well as activate the nuclear‐cytoplasmic shuttling to regulate a nuclear phosphoinositide cycle 1. RalA and RalB also interact with phospholipase D (PLD) which is involved in multiple cellular pathways 62. PLD1 appears to be present in the Golgi apparatus, endoplasmic reticulum, and late endosomes, whereas PLD2 seems to be associated with the plasma membrane and caveolae 63. PLD produces intracellular lipid messengers (PA) by hydrolyzing phosphatidylcholine which subsequently is involved in Golgi vesicle secretion and transport from the endoplasmic reticulum to the Golgi 64. The Ral–PLD complex regulates EGF receptor endocytosis 65 and it is believed that Arf1 is required for this complex to stimulate both PLDs 66. Furthermore, PLD activity is commonly increased in response to oncogenic and mitogenic stimuli which could be induced by Src and Ras 67.

The ZO‐1‐associated nucleic acid‐binding protein (ZONAB) has been recently described as a Ral effector that promotes cell proliferation and represses differentiation 68, 69. It is now clear that ZONAB functions are regulated by interaction between RalA and ZONAB in a cell density‐dependent manner 70. A model suggested by Frankel et al. (2005) indicated that RalA can control the transcriptional activity of ZONAB which is able to regulate gene expression. This may provide evidence showing the important contributing role of Ral to oncogenesis by Ras 1, 70. Although RalA and RalB have identical effector‐binding domains, differences in their ability to bind specific effector proteins and subcellular localization allow them to carry out unique functions and exhibit different roles in normal and neoplastic cell function 71.

## Ral Signaling in Normal Physiology

Substantial evidence now supports important physiological roles of Ral in normal cell function. The Ral pathway has been shown to be a potent player in regulating various transcription factors. Activation of the tyrosine kinase Src by the Ral‐GEF/Ral signaling pathway leads to the activation of transcription factor Stat3 and JNK kinase. This signal later activates c‐jun kinases, a major component of the AP‐1 transcription factor family, which stimulates transcriptional activity, cell differentiation, and apoptosis 72, 73, 74. Ral‐mediated phosphorylation of AFX, a mammalian‐related Forkhead transcription factor, is induced by PKB at T28, S193, and S258 75. Ral can also alter other transcription factors, including TCF, and NF‐kB. However, their exact effector proteins and pathways are not well understood.

It is mentioned that the expression of RLIP76 protein was discovered in all normal tissues, but expression varied depending on the tissue 76. RLIP76 binds to its protein‐binding partners, including AP2, POB1, Reps1, epsin, and Rab11‐FIP2 [reviewed in 71]. Furthermore, it seems that RLIP76 activation functions in response to oxidative stress in normal cells 77. Cellular studies also demonstrate that Ral‐GTP‐binding proteins are expressed in synaptic vesicles, platelet dense granules, and electromotor neurons 78, 79, 80, 81. Along with this, it has been illustrated that the Ral‐GTPase signaling pathway is associated with cell‐cycle G_1_‐ to S‐phase progression and stimulation of cyclin D1 gene transcription 82, 83, 84. Recently, research has shown that Ral could function as an important molecule playing a significant role in the differentiation and self‐renewal of hematopoietic cells 85. However, the exact physiologic function of the Ral signaling pathway and its downstream effector proteins are still unknown.

## Ral Signaling Pathways in Immune System

The Ral‐GTPases (RalA and RalB), directly or indirectly, appear to be implicated in triggering diverse immune‐signaling pathways. Both RalA and RalB have been shown to play crucial roles in lymphocyte function through cell‐mediated cytotoxicity in natural killer (NK) cells 9, 86. The proteins involved in Ras/Ral signaling initiate phosphorylation of threonines 447 and 451 which actively regulate AFX (acute‐lymphocytic‐leukemia‐1 fused gene from chromosome X) 75. It has also been previously demonstrated that AFX is directly controlled by the PI(3)K‐PKB pathway in response to insulin only on serines 193 and 258 87. In the Ras‐Ral pathway, activated RalA triggers granule polarization and secretion in a Sec5‐independent pathway; however, activation of RalB regulates only the granule secretion 86, 88. Both RalA and RalB were found to be associated with secretory lysosomes (cell type dependent) with different functions 89, 90. The involvement of Ral in the regulation of platelet dense‐granule secretion via inhibition of Sec5‐RBD is further supported by Kawato and coworkers 91. Gorter et al. have reported a novel function for Ral that is involved in the regulation of BCR‐induced gene transcription by controlling AP‐1 and NFAT activity, thereby providing new possibilities for Ral activity in B‐cell development and function 92. Gorter et al. have also demonstrated that Ral is activated in response to the stimulation of B cells and multiple myeloma (MM) cells with stromal cell‐derived factor‐1 (SDF‐1)‐controlled migration. This result may show the role of Ral in interfering with the control of SDF‐1–induced migration of B cells and MM cells 93.

Under the condition of virus‐induced stimulation, RalB and Sec5 comprise a complex, recruit the innate immunity‐signaling kinase TBK1, and trigger the IRF‐3 pathway leading to induction of interferon. Meanwhile, it is emphasized that the RalB/TBK1 pathway may activate an inflammatory response which can contribute to tumor promotion 94. RalBP1, a main effector of Ral, is associated with various immune‐mediated diseases. In fact, the C‐terminal of RalBP1 is responsible for immunoreactivity 95. Likewise, Ral binds to Sec5 and Exo84, and subsequently recruits exocyst function that is necessary for different cellular pathways such as polarized exocytosis, cytokinesis, cell migration, ciliogenesis, and wound healing 96, 97. Similarly, it has been demonstrated that neutrophil differentiation is regulated by the interaction of phospholipase D1 with Ral 98. In addition, the complex of RalA and PLD was found to be involved in the regulation of Fc*γ*R‐mediated phagocytosis 99. It appears that knowing details about exocyst structure and function, as well as their relative components by using a systemic biology approach for the prediction of exocyst components as regulators of phagocytosis, will likely provide understanding of many aspects of innate and adaptive immunity 100.

## Ral Signaling in Cancer

The oncogenic effects of Ral were discovered over two decades ago, expanding investigation into the tumorigenic role of Ral in a variety of different cancer types 101. The chronic activation of RalA and RalB was frequently reported by other studies in various tumor‐derived cell lines versus nontumorigenic counterparts, which supported the functional significance of Ral proteins in cancer 102, 103, 104, 105, 106. Using RNA inhibition (RNAi) of Ral protein expression, the role of Ral in cancer initiation and development was further supported 9, 107. Rals regulate tumorigenesis and cancer progression in three ways: (1) through activation of Ral effector proteins such as RalBP1 and kinase Aurora A, (2) via activation of several signaling pathways such as phosphaolipase D1, Src, JNK, NF‐kB, and cyclin D, and (3) by phosphorylation of Ral proteins 9, 13, 15, 102. Ral activation was shown to be involved in a number of different tumor types such as lung 105, colorectal 108, melanoma 109, 110, pancreatic 111, squamous cell carcinoma 112, hepatocellular carcinoma 113, 114, prostate 115, ovarian 116, bladder 12, chronic myelogenous leukemia 117, peripheral nerve sheath tumors 104, and medulloblastoma 118. Studies have even been completed showing Ral‐A autoantibodies as a potentially useful serum biomarker for prostate adenocarcinoma 119.

Both RalA and RalB are multifunctional proteins in the cancer milieu that are responsible for regulating tumor initiation, invasion, migration, and metastasis 3, 37, 105, 120, 121. Ral‐GTPases have been shown to play a role in prostate cancer metastasis 106. RalA and RalB collaborate to support pancreatic tumorigenicity through the regulation of tumor cell growth, invasion, and metastasis 102, 103. The loss of bone metastatic activity was found in prostate cells that have shRNA‐mediated inhibition of RalA expression 115. Failure in pancreatic metastasis was demonstrated by Lim's group (2005) in cells expressing RalB‐specific shRNA through chorinic RalB depletion. This group also observed that RalA is activated in the early stage and RalB is activated in the late stage of pancreatic tumorigenesis 102. RalA mRNA expression was reported to be enhanced in advanced stages of cancer. However, RalA mRNA expression was not significantly associated with higher tumor grade in patients with bladder tumors. Interestingly, the expression of both RalA and RalB protein levels was elevated in states of metastasis 122.

RalA plays a key role in RalGEF‐induced transformation and tumorigenesis of human cells 102. On the other hand, knockdown of RalA protein suppresses Ras activation or RalGEF‐induced anchorage‐independent growth 3, 102, 123. RalB seems to be more potent than RalA in the regulation of cell invasion and migration 103. Studies have shown that RalA plays an integral role in disrupting cellular adhesion via controlling Arf6 activation 124. Others have suggested RalA inhibits p53, which in turn helps promote invasion 125. The knockout of RalB leads to a decrease in the survival of transformed cells, cell motility, cell migration, and cell invasion 3, 123. RalA and RalB are known to play a role in metastasis, but RalA has been postulated to play a limited role 103. These experiments were performed in pancreatic, bladder, prostate, breast, liver, and NSCLC cells using RNAi 12, 103, 105, 106, 113, 126. Besides using genetic methods, it has been recently shown that the deletion of either RalA or RalB could not alter the formation of KRAS^G12D^‐driven tumors in a mouse model of NSCLC 127. A similar result was observed for DMBA‐TPA‐induced skin carcinogenesis in a mouse model of KRAS^G12D^‐driven pancreatic adenocarcinoma 127. It is assumed that the discrepancy between these results and prior reports may be caused by differences in the experimental methods 127.

Studies using a mouse model indicated that RalGDS is a putative effector molecule for Ras‐driven transformation and oncogenesis 128, 129. Activated versions of RalGDS have been previously shown to promote the metastasis of prostate cancer cells 130. It was then found that inhibition of RalGDS activity can reduce the incidence of papillomas which leads to less aggressive behavior of skin cells in vivo 129. In line with the previous findings, the importance of RalGEF‐Ral signaling has been seen in different stages of pancreatic ductal adenocarcinoma (PDAC) 103. Recently, RalGDS activity was reported to be required for progression of colorectal and melanoma tumors 108, 109, 110. Ral even appears to be required for promotion of tumorigenesis in tumors lacking Ras mutations 104. Zipfel's team has demonstrated that the Ral signaling pathway can be activated in melanoma in the absence of an oncogenic Ras mutation 110. Falsetti et al. (2007) have shown that geranyl geranyl transferase 1 inhibitors (GGTI) can abolish Ral function in pancreatic cancer cell lines in a Ras‐independent manner 123.

RalA was recently found to be phosphorylated on Ser11, Ser183, and Ser194. Changing phosphorylation sites could modulate the ability of RalA to associate with and regulate its downstream targets 11, 12, 13. By using alanine substitution A*β* mutants, Ser183 and Ser194, were shown to be important requirements for the interaction of RalA and PP2A A*β*, suggesting the importance of RalA in transformation associated with loss of functional A*β* in human cancers 13, 15. It has also been recognized that the phosphorylation of RalA at Ser183 by PKA and Ser194 by Aurora A might represent a RAS‐independent mechanism of Ral regulation 131. Similarly, the phosphorylation of RalB protein occurs on Ser198 by the kinase PKC which demonstrates the tumorigenic role of RalB through the promotion of internalization of the protein 12. Thus, the differences in posttranslational modification in RalA and RalB might be involved in distinct subcellular localizations, leading to the activation of certain effectors involved in malignant transformation 132. A recent report also explained the regulating role of extracellular superoxide dismutase (SOD3) on expression of RalGEF‐RGL1 in anaplastic thyroid cancer cells 133.

## Mutations in Ral Gene

In 1986, Chardin screened *λ*gt10 cDNA library with a 20‐mer oligonucleotide probe which corresponded to seven amino acids that were extremely preserved in all Ras member proteins from yeast to humans. He described a new Ras‐like gene which is known as Ral 5. Ral and Ras have 58% sequence homology with similar structural features 134. The most similar sequence between them was in the guanine nucleotides region 135. Oncogenic mutations in Ral's guanine nucleotides region can suppress its GTPase activity. Therefore, the survival signal will be constitutively transmitted to the cell and make Ral proteins resistant to GTPase‐activating protein (GAP) 2. A study on human bladder cancer reported one missense mutation of glutamine for glutamate at position 97 in the UM‐UC‐6 cell line, which is a G‐to‐C transversion at nucleotide position 589, but it did not show any higher GTP‐bound activity 122. It should be mentioned that Ral activity might be affected by mutations of G23V and Q27L in Ras gene codons 12 and 61 106.

Ral genes are responsible for RalA and RalB expression and they are downstream effectors of Ras 11. Although RalA and RalB have about 80% similarity in sequence, they are involved in different functions, which is primarily due to a 30 amino acid hypervariable region in the C‐terminal 102, 103. These hyper variable regions have a conserved CAAX motif which is targeted for protein prenylation 136. Targeted mutational analysis demonstrated that these regions have several phosphorylation sites that are exclusive for RalA and RalB 13. The RalA C‐terminus has two serine residues (S183 and S194) which are its unique phosphorylation sites and the RalB C‐terminus has five threonine or serine residues (T178, S182, S192, S193, and S198) which can undergo phosphorylation 10. Aurora A is a serine/threonine mitotic kinase that possesses a key role in mitosis entry, centrosome duplication, and cytokinesis 14. This kinase is mainly localized in the centrosome at different phases of mitosis and could be involved in tumorgenicity 137. S194 in RalA is dephosphorylated by PP2A 13, but a mutation of S194 to alanine or dephosphorylation with PP2A inhibits the transforming ability of RalA 15.

Ral is activated through several mechanisms, including overexpression or mutations of receptor tyrosine kinases 138. Furthermore, interactions between Ral and RalGEF can cause Ral activation.

## Oncolytic Viruses and Ral Signaling

Oncolytic viruses are a promising class of replication competent viruses that in many cases, such as herpes model, have passed phase III clinical trials 139. Reovirus is a benign human virus that has been under significant investigations including clinical trials as a cancer therapy agent 140, 141, 142. Oncolytic virus therapy is based on the ability of viruses to effectively infect and kill tumor cells without harming the normal tissues. While some viruses seem to have a natural preference for tumor cells, most viruses require the modification in their tropism to specifically enter and/or replicate in cancer cells 143, 144, 145, 146, 147. Permissiveness of cells to reovirus (a RNA oncolytic virus currently in advanced clinical trials 148, 149) has been shown to be dependent on RalA signaling 150. This was achieved by studying the permissiveness of RasV12G37 mutant (which is only capable of signaling through RalGEFs) to reovirus. Additionally, inhibiting RalA by a dominant negative mutant induced resistance to reovirus.

Brain tumors are considered one of the primary targets for oncolytic virus therapy including reovirus therapy 151, 152, 153. Malignant primary brain tumors infiltrate the brain tissue both near and far from the tumor. Effective treatment necessitates a model that can discriminate malignant cells from normal tissue and then selectively kill those cells. Oncolytic viruses represent a model that will allow treatment of the tumor without affecting the rest of the brain. Our recent unpublished work shows that RalA is overactivated in glioma and medulloblastomas, which makes them promising targets for reovirus therapy.

## RalA Signaling and Cancer Stem Cells

**Figure 3:**
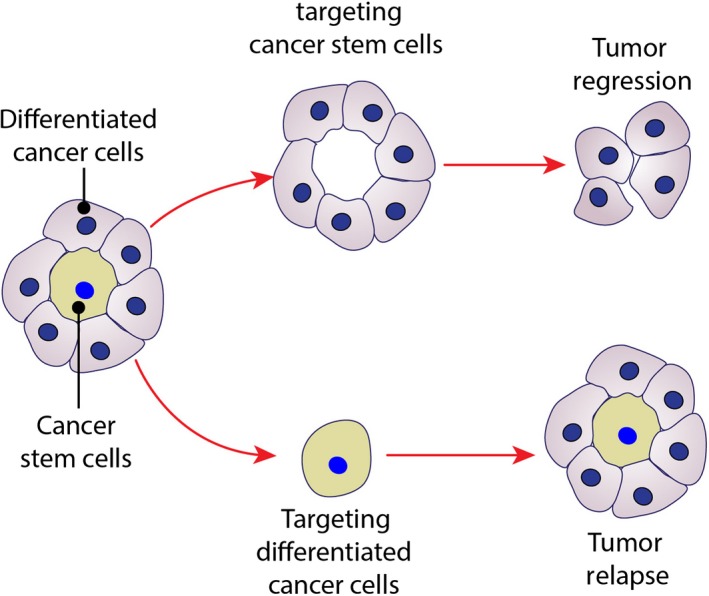
Cancer stem cells and their role in cancer therapy. New concepts in cancer biology define tumors as a highly heterogenic composition of different cells. From developmental point of view, tumors are made of cancer stem cells (CSCs) and differentiated cancer cells. CSCs are capable of producing different cells that are needed to maintain tumor including differentiated cancer cells. CSCs are resistant to conventional therapies and, therefore, if not eradicated, they can repopulate the tumor and case a relapse. Remedies that target CSCs offer a better solution for inducing significant tumor regression as differentiated cancer cells have a limited lifetime.

New findings in cancer biology emphasize the heterogenic nature of tumors. The biology of these cells, including their cell signaling machinery, can differ widely, resulting in their varying sensitivity to chemotherapy. Two major categories of tumor cells include cancer stem cells (CSCs) and differentiated cancer cells. CSCs constitute a minority population with enhanced tumorigenesis, invasiveness, and resistance to apoptosis (Fig. 3) 154, 155, 156. Progression of tumors is usually correlated with higher percentage of cancer stem cells in the tumor cell population 157, 158. Among the few methods for detecting cancer stem cells, such as functional assays (e.g., ALDH1 assay) or dye exclusion assays (dye cycle violet assay), antibody‐based detection and sorting of surface markers (e.g., CD133, CD24, CD44, etc.) is a popular method 159. The resistance of CSCs to conventional therapy and their ability to repopulate tumors necessitates a better understanding about their cell signaling features.

Interestingly, it was shown that in cancer stem cells, a(v)b3 integrin recruits RalB and KRAS to the plasma membrane resulting in the induction of TBK1 and NF‐kB activation which contributes to the refractory status to erlotinib, a tyrosine kinase inhibitor 160.

Our team was interested in evaluating the level of RalA activation in CSC in comparison to differentiated cancer cells. In ovarian cancer cell lines, CD24 has been determined to serve as a marker for cancer stem cell population 158, 161. Thus, we evaluated the level of RalA activation in CD24 high populations relative to CD24 low populations, as expression of this marker seems to follow a spectrum in ovarian cancer cells. We sorted these populations by flow cytometry and found a marked increase in RalA activation in CD24 high population 29. Given that CD24 is a proven marker for cancer stem cells in ovarian cell populations, CD24 could provide a novel target for therapy.

RalA activation in lung cancer stem cells (LCSCs) was analyzed as well, specifically in CD44 cells, a known marker for LCSCs 105, 162, 163. A549 (adenocarcinoma) cell population was sorted via flow cytometry according to high (CD44 Bright) and low CD44 (CD44 Dim) expression 105. Once sorted, RalA activation was analyzed in these two cell populations. The CD44 Bright population had elevated RalA activation relative to the CD44 Dim population, suggesting RalA activation might play a major role in CD44+ cell biology. Again, this could provide a target to not only eradicate differentiated cancer cell populations but also CD44 cancer stem cell populations as well.

We also wanted to analyze RalA activation in cancer stem cell populations in hepatocellular carcinoma (HCC) 28. As CD133 has been suggested to be a marker for HCC cancer stem cells, we analyzed expression of CD133 in different HCC cell lines 164, 165. We then looked at RalA activation as a function of CD133 expression and found a direct correlation between CD133 expression and RalA activation. Also, once HCC cells were enriched for CD133 +  cells, a higher level of RalA‐GTP was observed 28. Similar results were observed when CD133+ cells were sorted from primary malignant peripheral nerve sheath tumor (MPNST) cell population 104, 166. While the level of RalA‐GTP was elevated in MPNST cells as compared with nonmalignant Schwann cells, the expression of RalA and its activation were found to be higher in CD133+ MPNST‐enriched cell population in comparison to CD133‐depleted population 166. Finally, medulloblastoma CD133+ cells were also found to contain higher levels of RalA activation 118.

We conclude from our studies that RalA plays a significant role in the biology of CSCs. This pathway provides an avenue for future research, especially as a possible explanation for why these populations are particularly resistant to therapy. The inhibition of this pathway could potentially serve as a novel therapeutic target in the CSC population.

## Therapeutic Approaches Targeting Ral in Cancer Therapy

As inhibition of the Ras pathway has not been a promising approach in cancer therapy, a possible alternative could be to block this pathway indirectly by targeting downstream effectors of Ras, including, RalGDS, Ral, and RalBP1 37. One of the most promising approaches is targeting the RalGEF signaling pathways focusing on the inhibitors involved in Ral posttranscriptional processing and effector signaling. Phosphorylated GTPases have an important role in cancer including apoptosis inhibition, cell growth induction, invasion, and angiogenesis 167, 168, 169. Additionally, protein kinases are appropriate targets for drug discovery. Inhibitors of GGTaseI inhibit growth in MIA‐PaCa2 cells which induce RalB‐dependent apoptosis and RalA cell‐cycle perturbation 123. It has been recognized that GGTI and Docetaxel, a chemo anticancer drug, have synergistic effects on the growth of prostate cancer cell lines including LNCaP, PC3, and DU145 169, 170. Currently, GGTI‐2418 is in phase I clinical trials and has shown minimal side effects (http://www.tigrispharma.com/).

Another interesting pattern in the regulation of G proteins is phosphorylation by kinases and their localization. Phosphorylation of Aurora A on S194 results in relocation of RalA from the plasma membrane to endomembranes where it associates with RalBP1/RLIP76 15. Aurora inhibitors may cause RalA to be selectively inhibited. There are several Aurora family inhibitors such as ZM447439 171, Hesperadin 172, and VX‐680/MK‐0457 173, which are competitors of ATP binding on Aurora molecules preventing the activation of Aurora family members. It has been established that VX‐680/MK‐0457 has higher specificity to Aurora A than two other inhibitors. However, Hesperadin has shown specificity to Aurora‐B 171, 172. Phosphorylation of S198 at the C‐terminal in RalB by PKC in T24 or UM‐UC‐3 bladder cancer cell lines can cause translocation of RalB from the plasma membrane to the perinuclear region. This induces anchorage‐independent growth, cell migration, and lung metastasis 12. Therefore, kinase inhibitors such as staurosporine might lead to inhibition of invasion and migration in human bladder carcinoma cell‐line EJ 174. Taken together, PKC inhibitors including Go6976 are considered effective molecules to inhibit metastasis due to the influence on cell‐to‐cell and cell‐to‐matrix junctions in bladder cancer 175. PCK inhibitors have also shown to be effective against malignant peripheral nerve sheath tumors in neurofibromatosis type I 176. In oncogenic stress situations, some apoptotic signaling pathways are activated. However, RalB binding to Sec5, which is a component of the exocyst, activates a typical IkappaB kinase family member TBK1. This kinase restricts initiation of apoptosis signaling pathways and couples with tumor cell survival 177. The cyclin‐dependent kinase 5 (CDK5) is linked to Ral function in a way that RalA and RalB activation and expression of Rgl2 are decreased through CDK5 inhibition. Thus, CDK5 inhibitors may act as RalA and RalB inhibitors in pancreatic cancer 178.

## Conclusion

Ral signaling pathway plays an important role in health and cancer. Overactivation of Ral and its signaling through important effectors such as RalBP1 portrays and opportunity for development of a novel class of anticancer drugs. Our work predicts that such anti‐RalA signaling agents will have enhanced effects against CSCs. Such characteristics would be of special importance as majority of our current anticancer agents lack significant activity against CSCs. Elucidation of detailed signaling components of this pathway can reveal new targets for treatment of human cancer 179, 180.

## Conflict of Interest

None of the authors has disclosed conflict of interest.
